# Tracking Canada’s 2015 vaccine research and development priorities: Where are we a decade later?

**DOI:** 10.14745/ccdr.v52i03a05

**Published:** 2026-03-31

**Authors:** Nasheed Moqueet, Kyle Lago, Serena Cortés-Kaplan, Harsimrat Birdi, Shalini Desai, Alisha Gauhar, Bryna Warshawsky, Matthew Tunis, Krista Wilkinson

**Affiliations:** 1 Centre for Immunization Surveillance and Programs, Public Health Agency of Canada, Ottawa, ON; 2 Department of Epidemiology and Biostatistics, Western University, London, ON

**Keywords:** human vaccines, infectious diseases, vaccine research and development, R&D, Canada, public health, prioritization

## Abstract

**Background:**

In 2015, Public Health Agency of Canada (PHAC) identified a set of priorities for research and development (R&D) of new and improved human and animal vaccines. Thirty human pathogens were grouped by vaccine development timeline (short: 0–6 years; medium: 7–12 years; long: 13 years or longer) and ranked by R&D priority (high, medium, low).

**Objective:**

To characterize the vaccine development pathway for these 30 pathogens to inform a 2025 update to PHAC’s vaccine R&D priorities.

**Methods:**

For each pathogen, we conducted a targeted search for vaccines authorized in Canada since 2015 using the Health Canada Drug Product Database and Canadian Immunization Guide and for candidates in clinical trials, in the registry, ClinicalTrials.gov (primary completion date of May 1, 2015 or later). Search results were downloaded and filtered by study status, phase and type. For select pathogens, we conducted additional searches in published (PubMed) and grey literature (other trial registries, industry press releases, and Web searches).

**Results:**

Seven pathogens had at least one newly authorized vaccine since 2015: three of 13 high-priority (influenza, n=4; *Streptococcus pneumoniae*, n=2; respiratory syncytial virus, n=3); two of eight medium-priority (herpes zoster, n=1; meningococcal serogroup B, n=1); and, two of nine low-priority pathogens (dengue, n=2; human papillomavirus, n=1). Nineteen pathogens had no authorized vaccine in Canada or elsewhere, although five had candidates in phase 3 trials (*Clostridioides difficile, Neisseria gonorrhoeae, Borrelia burgdorferi*, norovirus and cytomegalovirus).

**Conclusion:**

Although some of the pathogens on the 2015 list now have authorized vaccines or candidates in late-stage clinical development, important gaps persist, which will inform PHAC’s 2025 vaccine R&D update.

## Introduction

In 2015, the Public Health Agency of Canada (PHAC, “Agency”) published a set of priorities for research and development (R&D) focusing on new and improved human and animal vaccines ((1)). The Agency consulted expert groups in a three-stage process to establish a final list of pathogens/diseases (“pathogens”) of low, medium and high-priority for vaccine R&D based on their public health impact in the Canadian context and framed within likely time horizons of a vaccine coming to market.

Since 2015, significant advances in technology and data science have led to substantial progress in vaccine development. The COVID-19 pandemic further accelerated vaccine development by enabling the widespread adoption of mRNA vaccine technology while highlighting the necessity of strengthening all stages of the vaccine life cycle ((2)). Moreover, the pandemic demonstrated that, with adequate resources and coordinated efforts, vaccine development timelines can be significantly shortened ((3)). During the pandemic period between 2020 and 2024, 45 vaccines were authorized in Canada (30 were for COVID-19 vaccines), which is considerably higher than the decades prior (2000–2009, 31 authorizations; 2010–2019, 34 authorizations) ((4)). It is unclear whether such timelines are sustainable in a non-emergency setting.

In light of the changing vaccine landscape, progress in vaccine development was examined for the 30 human pathogens on the 2015 R&D priority list, the majority (n=21, 70%) of which did not have an authorized vaccine in Canada in 2015. A more detailed examination was conducted of vaccines targeting high-priority pathogens, as well as those in advanced stages of clinical testing (i.e., at phase 3) for medium- and low-priority pathogens. The primary aims in mapping pathogens designated as priorities in 2015 to the current vaccine landscape are to generate evidence to inform the 2025 update to PHAC’s vaccine R&D priorities and to provide strategic guidance to investigators engaged across different phases of the research lifecycle.

## Methods

First, vaccines authorized in Canada for each human pathogen were identified using the Canadian Immunization Guide ((5)) (listed under the section “Preparations authorized for use in Canada”) and the Health Canada Drug Product Database ((6)). Then, for pathogens lacking authorized vaccines in Canada, the United States (US) clinical trials registry, ClinicalTrials.gov ((7)), was searched for vaccine candidates still undergoing testing. Targeted searches of published literature were also conducted in PubMed, in addition to grey literature searches across other databases and select clinical trial registries. Details on data sources, search parameters and date limits are provided in the **Appendix**, Table A1 ((8)).

## Results

**Figure 1:**
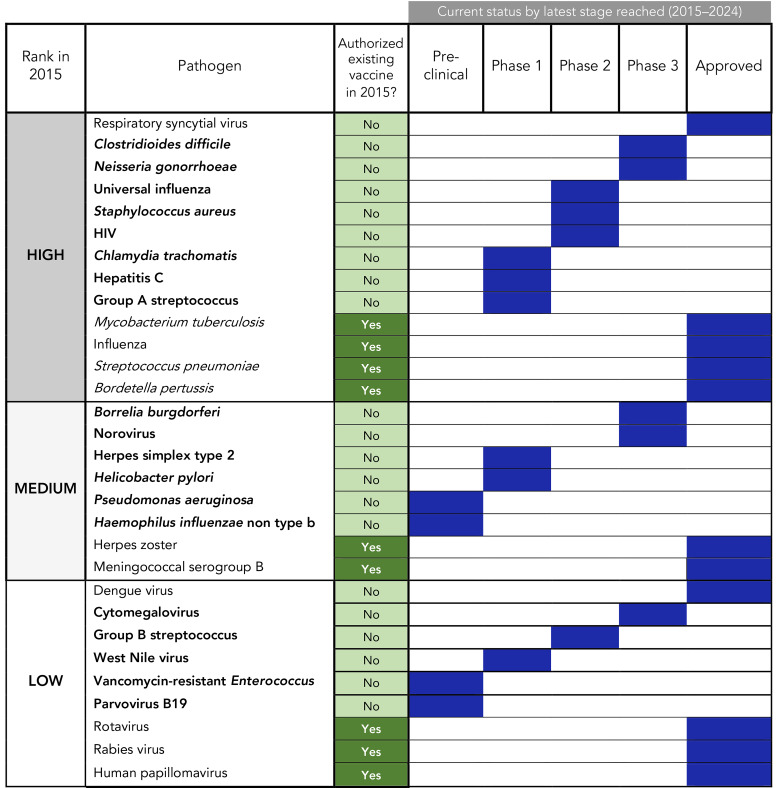
Status of vaccine development of all human pathogens from the 2015 research and development (R&D) list as of 2024^a,b,c,d,e^ ^a^ Pathogens shown in bold are those that lack authorized vaccines in 2024 (possible candidates for 2025 list) ^b^ “Authorized existing vaccine in 2015” refers to status in Canada in 2015: No=new vaccine required; Yes=existing vaccine could be improved ^c^ ”Approved” as a “Current status” refers to any approval for human use in Canada or outside Canada in 2015 or later. Those with existing vaccines in 2015 are all marked “approved” ^d^ For *Mycobacterium tuberculosis*, earliest year of marketing of the Bacille Calmette-Guérin (BCG) vaccine in Canada is 1961, as noted by Health Canada’s Drug Product Database. Though the Canadian Immunization Guide lists the BCG vaccine as a “Preparation authorized for use in Canada,” most provinces and territories discontinued routine use in the 1970s; however, BCG is part of the publicly funded childhood vaccination program in the Northwest Territories and Nunavut; in other parts of Canada, it is also used for tuberculosis prevention in infants in high risk communities. The BCG vaccine may also be considered in exceptional circumstances, such as for persons at high risk of repeated exposure, for certain long term travellers to high prevalence countries and in infants born to mothers with infectious tuberculosis disease ^e^ Results for *Haemophilus influenzae* non type b are from the last search on May 2024. A phase 1 trial (NCT06465420) was registered in ClinicalTrials.gov in June 2024, which was after the last search was conducted

**Figure 2:**
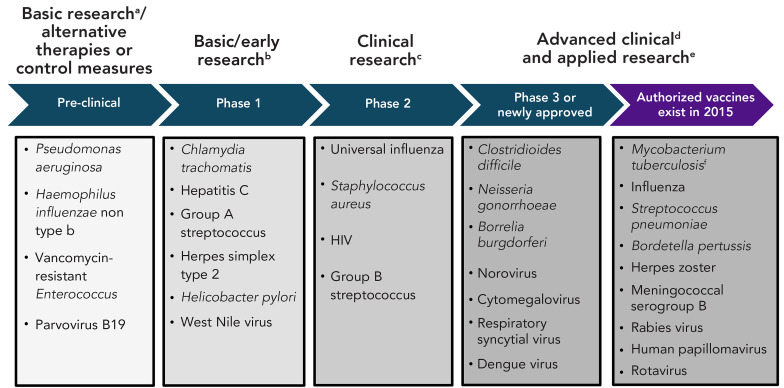
Human pathogens from the 2015 research and development (R&D) list grouped by most advanced vaccine development stage to guide future research efforts^a,b,c,d,e,f^ ^a^ “Basic research” in “Basic research/alternative therapies or control measures” refers to focusing on laboratory and animal testing. Sample research questions can address basic biology or immunology (e.g., characterizing pathogen biology, transmission dynamics and host immune responses, identifying novel antigens and vaccine targets, developing and validating animal models for vaccine testing). Main stakeholders include basic science or academic research institutes or wet lab scientists ^b^ “Basic/early research” focuses on safety, preliminary immune response and dosage in humans. Sample research questions can focus on immunology and vaccine design (e.g., proof-of-concept studies to validate immunological mechanisms and immune response, optimizing vaccine formulations and dosages, adjuvants and delivery platforms, investigating immune escape mechanisms and antigenic variation to inform vaccine design). Key contributors are typically academic or government research institutes, early-stage biotech companies and manufacturers ^c^ “Clinical research” refers to a focus on expanded safety and immunogenicity. Sample research and development (R&D) questions can focus on immunological performance of the vaccine in targeted or general populations (e.g., immunogenicity studies, dose and schedule optimization studies), as well as ongoing safety monitoring. Key contributors include clinical research networks, academic medical centers and manufacturers ^d^ “Advanced clinical” in “Advanced clinical and applied research” refers to research focusing on establishing efficacy and safety in large clinical trials along with assessments of vaccine quality required for authorization (phase 3 and onwards). Large efficacy studies are most often led by manufacturers ^e^ “Applied research” in “Advanced clinical and applied research” refers to research focusing on implementation and post-market surveillance: questions address programmatic factors (e.g., assessing acceptability and barriers to uptake, optimal delivery mechanisms, cost-effectiveness) and post-licensure surveillance (e.g., ongoing monitoring of effectiveness, safety in real-world settings). Post-authorization studies can also address vaccine response and safety in special populations not previously studied in clinical trials (e.g., people who are pregnant, immunocompromised or children), concurrent administration with other vaccines, alternate schedules and duration of protection. Routine post marketing surveillance are best addressed by federal/provincial/territorial health authorities. Observational effectiveness studies are best addressed by research networks or institutes of academic partners ^f^ For *Mycobacterium tuberculosis*, though the Canadian Immunization Guide lists the Bacille Calmette-Guérin (BCG) vaccine as a “Preparation authorized for use in Canada”, most provinces discontinued routine use in the 1970s. However, BCG is part of the publicly funded childhood vaccination program in the Northwest Territories and Nunavut; in other parts of Canada, it is also used for tuberculosis prevention in infants in high risk communities. The BCG vaccine may also be considered in exceptional circumstances, such as for persons at high risk of repeated exposure, for certain long term travellers to high prevalence countries and in infants born to mothers with infectious tuberculosis disease Note: Groups correspond to the most advanced stage reached as of 2024 and serve as broader, illustrative themes. They are not intended as exhaustive or rigid classifications. Specific R&D questions, stakeholder roles and study designs frequently vary or overlap based on vaccine-specific and pathogen-specific factors. Examples of R&D questions and key stakeholders at each stage are in footnotes above

Since 2015, two pathogens (respiratory syncytial virus [RSV] and dengue virus) from the 2015 R&D list, which did not have existing authorized vaccines, had newly authorized vaccines for human use, though only RSV vaccines received approval in Canada. Based on publicly available information as of April–May 2024, 19 pathogens still had no authorized vaccine in Canada or elsewhere, though five had candidates in phase 3 trials (*Clostridioides difficile, Neisseria gonorrhoeae*, *Borrelia burgdorferi*, norovirus and cytomegalovirus [CMV]). **Figure 1** presents updates on all 30 human pathogens by the most recent research stage achieved as of 2024, while **Figure 2** highlights the potential implications of this progression for R&D across the research lifecycle. The following sections also provide comprehensive summaries of the most advanced candidates of the high-priority pathogens that lacked authorized vaccines in Canada in 2015. For medium- and low-priority pathogens, a detailed overview is provided for phase 3 or newly authorized vaccine candidates only. Brief updates on all pathogens with existing authorized vaccines in 2015 are provided in the Appendix ((8)).

### High-priority pathogens: Overview

Of the 13 high-priority pathogens on the 2015 list, among those that had existing authorized vaccines in Canada, there were new approvals for influenza and *Streptococcus pneumoniae*, phase 3 candidates for tuberculosis (TB) and label changes for *Bordetella pertussis*. In addition, RSV, for which there was no vaccine in 2015, received its first Canadian authorization in 2023. Among the remaining eight high-priority pathogens, two had at least one candidate in phase 3 testing (*C. difficile, N. gonorrhoeae*), three were in phase 2 (*Staphylococcus aureus*, HIV, universal influenza) and three were in phase 1 (Group A streptococcus, hepatitis C, *Chlamydia trachomatis*) (Figure 1).

Details of the most advanced candidates are described below for the nine high-priority pathogens lacking an authorized vaccine in 2015. Additional information for all the high-priority pathogens is available in the Appendix ((8)).

High-priority pathogens: Status for those lacking authorized vaccines in 2015 in Canada

**Respiratory syncytial virus (RSV):** Three vaccines for RSV have been authorized since 2015: two subunit vaccines containing a stabilized prefusion confirmation of the RSV F glycoprotein (ABRYSVO® by Pfizer and AREXVY® by GlaxoSmithKline [GSK]); and one mRNA vaccine encoding the RSV F glycoprotein (mRESVIA® by Moderna). In Canada, all three vaccines were approved for older adults for the prevention of RSV-associated lower respiratory tract disease, while ABRYSVO was also approved for pregnant individuals to protect infants from birth to six months of age against RSV-associated lower respiratory tract disease.

***Clostridioides difficile*:** One toxoid-based vaccine candidate for *C. difficile* was tested in a phase 3 trial in older adults (NCT03579459, NCT03090191, NCT03918629). In their most recently reported data, Pfizer’s PF–06425090 did not meet its primary efficacy endpoint (prevention of primary *C. difficile* infection), though the candidate was deemed safe and well tolerated and showed potential clinical benefit by reducing severe outcomes like *C. difficile* infection requiring medical attention ((9)). Pfizer is currently evaluating next steps for this vaccine ((10)).

***Neisseria gonorrhoeae*:** We identified two platforms for *N. gonorrhoeae* vaccine candidates currently in active phase 3 trials ((8)). Developed by GSK using a Generalized Modules For Membrane Antigens (GMMA)-based platform, NgG is the only candidate to specifically target *N. gonorrhoeae*, as all other trials are investigating whether an existing meningococcal B vaccine, 4CMenB (Bexsero), is effective in preventing *N. gonorrhoeae* infection. NgG is currently in phase 1–2 trials (NCT05630859) and being tested in healthy adults aged 18–50 years. One phase 2 trial of Bexsero (NCT04722003) is being conducted in both male and female adults, while all other trials (NCT04415424, NCT05294588, NCT05766904) have sex/gender restrictions, involving only males, nonbinary individuals, trans women or those assigned male at birth. Both phase 3 trials of Bexsero (NCT04415424, NCT05766904) had listed completion dates in 2025.

**Universal influenza:** Five candidate universal influenza vaccines (OVX836, chimeric hemagglutinin (HA) construct, M2SR, INFLUENZA G1 and FLU-v) meeting our eligibility criteria had completed phase 2 trials. Developed by Osixav, OVX836, is a novel recombinant universal influenza vaccine candidate based on a nanoparticle platform that targets the internal nucleoprotein. In its phase 2 trial (NCT05060887), OVX836 appeared to be safe and well-tolerated, eliciting humoral and cellular nucleoprotein-specific immune responses and a preliminary signal of protection against influenza ((11)). Both chimeric HA construct and M2SR are based on influenza virus-based platforms and have completed phase 2 trials in healthy adults (EudraCT 2017–001584-20, EudraCT 2017–004971–30, respectively), as have INFLUENZA G1 (NCT05901636) and FLU-v (NCT03180801, NCT02962908), which both use recombinant-based platforms. While results from some of these trials are still being reported, future activities and progression to phase 3 testing are unknown.

***Staphylococcus aureus*:** In the past decade, several candidates using diverse platforms and antigens from *S. aureus* have been tested but none has been successfully authorized. As of May 2024, two candidate vaccines have reached phase 2 in different settings and for different outcomes: rFSAV, a recombinant five-antigen vaccine was tested in elective surgery patients in China (Chi CTR2200066259) ((12)) after completing phase 1 trials (NCT02804711 and NCT03966040); and Biomed rTSST–1, a recombinant toxic shock syndrome toxin-1 variant vaccine, completed its phase 2 trial in January 2021 in healthy adults for prevention of staphylococcal toxic shock syndrome (NCT02814708) ((13)). Both vaccines were reported to be safe, well-tolerated and immunogenic in their target populations, though future testing activities are unclear.

**Human immunodeficiency virus (HIV):** Of the 66 potential trials identified from the general and targeted search for HIV vaccine candidates, only two were considered relevant and both were at very early stages (phase 1/2a) of testing in adults. The HIV–CORE0051 study utilizing a chimeric T cell epitope insert (HIVACAT T-cell immunogen or HTI) with sequential administration of a replicative defective chimpanzee adenoviral vector (ChAdOx1) and Modified vaccinia Ankara (MVA) vector, completed phase 1/2a testing in August 2022 (NCT04563377). VIR–1388 (NCT05854381), utilizing a CMV vector, will be tested in phase 1 trials in adults living with asymptomatic CMV. Two other candidates (Ad26.Mos4.HIV, ALVAC-HIV) which were tested in healthy adults in phase 2b/3 trials were both discontinued for failing to protect against HIV acquisition ((14,15)).

***Chlamydia trachomatis*:** *C. trachomatis*, which was at a pre-clinical stage in 2015, had one candidate, CTH522, which completed phase 1 testing (NCT02787109) in female adults aged 18–45 years. A recombinant protein subunit vaccine, CTH522 was tested for safety and immunogenicity in unadjuvanted or adjuvanted (either with cationic liposomal adjuvant CAF01 or aluminium hydroxide) forms. Both adjuvanted forms were found to be safe, well-tolerated and immunogenic, although CTH522/CAF01 had a better immunogenicity profile ((16)). Plans for future testing and development are unclear.

**Hepatitis C virus (HCV):** Hepatitis C virus, which had candidates in phase 2 testing in 2015, had only one candidate that met our eligibility criteria. AdCh3NSmut/MVA-NSmut completed phase 1 trials in various study populations (NCT02568332, NCT02362217, NCT03688061, NCT01296451). This vaccine used a sequential, heterologous prime-boost vaccination regimen based on a replicative defective chimpanzee adenoviral vector and MVA vector encoding non-structural proteins (NS3, NS4, NS5A and NS5B) of HCV genotype–1b. In its phase 1 trial, AdCh3NSmut/MVA-NSmut generated very high levels of both CD8+ and CD4+ HCV-specific T cells targeting multiple HCV antigens ((17)); however, results from a phase 2 trial in people who inject drugs (NCT01436357) failed to demonstrate protection against chronic HCV infection, although there was some virological evidence of partial HCV control in vaccine recipients (i.e., lower peak HCV RNA levels during acute infection in vaccine recipients vs. placebo) ((18)). Future testing activities related to AdCh3NSmut/MVA-NSmut are unclear.

**Group A streptococcus:** As of May 2024, Group A streptococcus had three vaccine candidates in phase 1 trials in healthy adults, all targeting antigens of the M-protein, a key virulence factor. StreptAnova is a 30-valent recombinant vaccine targeting M-proteins found on the surface of 30 Group A streptococcal serotypes. In a phase 1 trial at Dalhousie University (NCT02564237), StreptAnova was reported to be well-tolerated and immunogenic and did not elicit autoimmune or cross-reactive antibodies ((19)). Another candidate, MJ8VAX, also demonstrated safety and immunogenicity in its phase 1 trial (ACTRN12613000030774) ((20)), while the phase 1 trial status of a related candidate, P*17/S2 combivax, is listed as “active, not recruiting” (NCT04882514) with an estimated completion date of December 2025.

### Medium-priority pathogens: Candidates undergoing phase 3 testing

Six medium-priority pathogens lacked authorized vaccines in 2015 and of these, two (*B. burgdorferi* and norovirus) progressed to phase 3 trials.

***Borrelia burgdorferi* (Lyme disease):** VLA15, a recombinant protein vaccine by Pfizer and Valneva, targets six *B. burgdorferi* serotypes and is currently undergoing a phase 3 trial among participants aged five years and older in North America and Europe in areas where Lyme disease is highly endemic (NCT05477524). This vaccine is also being tested separately for safety in a phase 3 trial in healthy children aged 5–17 years in the US (NCT05634811).

**Norovirus:** Of the six unique vaccine candidates for norovirus that met our eligibility criteria, two have progressed the furthest and are being tested in study populations of young children. HIL–214 utilized a 2-dose bivalent virus-like particle formulation and is undergoing phase 2b/3 testing (NCT05281094) in children aged five months, while another candidate, Human Norovirus Bivalent (G I.1/G II.4) vaccine, a bivalent recombinant vaccine, is in phase 3 testing (NCT05916326) in children aged six months to 13 years.

### Low-priority pathogens: New authorizations or undergoing phase 3 testing

Six of the low-priority pathogens lacked any authorized vaccines in 2015 and, of these, two demonstrated progress, either by receiving authorization in other jurisdictions outside of Canada (e.g., dengue) or by reaching phase 3 trials (e.g., CMV).

Dengue vaccines which received authorizations outside Canada include Sanofi’s Dengvaxia®, approved in the US in 2019 ((21)) and Takeda’s Qdenga®, which was authorized in the European Union in 2022 ((22)). Both these vaccines are designed to protect against all four serotypes of the dengue virus (DENV–1, DENV–2, DENV–3 and DENV–4); however, the authorized age ranges differ, with Qdenga indicated for individuals as young as four years old and Dengvaxia indicated for those aged nine years and older. Dengvaxia is also not recommended for dengue-naive individuals, while Qdenga can be given regardless of prior infection or exposure history.

For CMV, only Moderna’s mRNA candidate, mRNA–1647, reached phase 3 testing (NCT05085366), though several other vaccines candidates were at earlier phases of development. mRNA–1647 was being tested for safety, immunogenicity and efficacy in preventing primary CMV infection among healthy women aged 16–40 years.

## Discussion

Vaccine development was monitored for the 30 human pathogens that were included in PHAC’s 2015 R&D list due to their associated disease burden, public health impact and/or concerns of antimicrobial resistance. Between 2015 and 2024, two pathogens (RSV and dengue) received their first-ever vaccine approvals (RSV in Canada and dengue in some other countries); however, 19 pathogens remain without a licensed vaccine in Canada or globally, underscoring ongoing gaps in vaccine R&D. Despite the limited number of new authorizations for the pathogens in the 2015 R&D priority list, progress was observed for five pathogens (*C. difficile, N. gonorrhoeae, B. burgdorferi*, norovirus and cytomegalovirus) that are now at phase 3 testing.

Progression through clinical phases is not linear. Though advances were noted among several high-priority pathogens that lacked vaccines in 2015 (RSV, *C. trachomatis*, *N. gonorrhoeae*), four actually regressed (i.e., were at an earlier clinical phase in 2024 compared to 2015: Group A streptococcus, HIV, hepatitis C and universal influenza). Of these, HIV and universal influenza were at phase 3 in 2015 but had receded to phase 2 in 2024, while Group A streptococcus and hepatitis C were at phase 2 testing in 2015, but at phase 1 in 2024. It is possible that some vaccine candidates identified in this paper will not proceed further along the pipeline; however, despite lack of progress in vaccine approvals for some high-priority pathogens since 2015, there were other notable non-vaccine achievements in prevention and disease control, especially for higher-risk priority populations, such as chemoprophylaxis for HIV ((23–25)), *C. trachomatis* ((26)) and *N. gonorrhoeae* ((26)) and highly curative treatments with shorter regimens for hepatitis C ((27)).

The Canadian vaccine R&D landscape is complex and multifaceted, requiring coordinated efforts among different stakeholders depending on the stage of development. To guide future research efforts, pathogens were grouped by their latest stage of development. It is worth noting that once human testing has begun, only 33.4% of candidates are successfully licensed ((28)), with the highest risk of failure (“valley of death”) lying between phases 2 and 3 ((29,30)), when primary outcomes expand beyond safety and immunogenicity (focus of phase 2) to include efficacy in larger study populations (phase 3). Pathogens at opposite ends of the development spectrum require distinct research approaches and stakeholders. Pre-clinical candidates require basic science investigations in academic settings to establish animal models and identify vaccine targets, while phase 3 candidates require large clinical trials for regulatory approval, ongoing post-market surveillance and applied implementation research for provincial/territorial authorities (details of setting, stakeholder and type of questions to address are shown in Figure 2 and its footnotes). By this reasoning, seven pathogens can be classified as requiring a focus on advanced clinical and applied research because they have candidates in phase 3 testing or have recent new authorizations, along with nine other pathogens that had existing vaccines in 2015. On the other end of the research lifecycle are those best suited to a focus on basic research or alternative therapies or control measures, which include the four medium or low-priority pathogens that had no candidates in clinical testing (i.e., were at the pre-clinical stage): *Haemophilus influenzae* non type b; *Pseudomonas aeruginosa*; Vancomycin-resistant *Enterococcus*; and parvovirus B19 (Figure 1 and Figure 2). Of note, all high-priority pathogens had at least one vaccine candidate in active clinical trials (phase 1 onwards).

### Strengths and limitations

Strengths of this study included a thorough, reproducible search of data sources most relevant to the Canadian context (Canadian registries or databases or those from the US or other similar high-income settings) supplemented with a targeted search of published literature and reports. The results of the quality crosscheck and careful review from multiple data sources also ensured the final results are accurate and complete. Nevertheless, because trial registries rely on self-report from investigators and results are limited by the specific search parameters applied, it is possible candidates included in the final results had been terminated or will not progress further or that relevant trials were registered after the search dates.

These inherent limitations of cross-sectional registry searches are partially offset by the continuous monitoring approach of multiple data sources (reports, registries, press releases and published literature) for priority pathogens to inform the 2025 R&D update. The requirement by regulatory agencies (Health Canada, US Food and Drug Administration) to register vaccine trials before reviewing for authorization also ensure that any significant progress for our pathogens of interest will be captured. It should be noted that because this work is very specific to the Canadian context, major international milestones (e.g., malaria vaccines) were not captured. Finally, while the 2015 R&D initiative mentions some pathogens with pandemic or outbreak potential (e.g., influenza, either as universal influenza or porcine influenza A), others like mpox and SARS-CoV-2 were not mentioned because they caused widespread outbreaks after the publication of the list.

## Conclusion

This study enabled the description and tracking of progress in vaccine development for pathogens in the 2015 Canadian R&D list, ensuring the 2025 update builds on past lessons rather than starting anew. Even though some of the pathogens now have authorized vaccines or candidates in late-stage clinical development, important gaps persist which will inform PHAC’s 2025 vaccine R&D update and have potential implications for investigators involved in different phases of the research lifecycle.

## Appendix

Supplemental material is available upon request to the author: nasheed.moqueet@phac-aspc.gc.ca or via the link included in reference 8.
